# Understanding Autoimmunity: Mechanisms, Predisposing Factors, and Cytokine Therapies

**DOI:** 10.3390/ijms25147666

**Published:** 2024-07-12

**Authors:** Farzana Yasmeen, Rameez Hassan Pirzada, Bilal Ahmad, Bogeum Choi, Sangdun Choi

**Affiliations:** 1 Department of Molecular Science and Technology, Ajou University, Suwon 16499, Republic of Korea; farzana892019@ajou.ac.kr (F.Y.); qhrma203@ajou.ac.kr (B.C.); 2 S&K Therapeutics, Ajou University Campus Plaza 418, Worldcup-ro 199, Yeongtong-gu, Suwon 16502, Republic of Korea

**Keywords:** autoimmunity, immune tolerance, targeted immune therapies, TNF, IL-1

## Abstract

Autoimmunity refers to an organism’s immune response against its own healthy cells, tissues, or components, potentially leading to irreversible damage to vital organs. Central and peripheral tolerance mechanisms play crucial roles in preventing autoimmunity by eliminating self-reactive T and B cells. The disruption of immunological tolerance, characterized by the failure of these mechanisms, results in the aberrant activation of autoreactive lymphocytes that target self-tissues, culminating in the pathogenesis of autoimmune disorders. Genetic predispositions, environmental exposures, and immunoregulatory disturbances synergistically contribute to the susceptibility and initiation of autoimmune pathologies. Within the realm of immune therapies for autoimmune diseases, cytokine therapies have emerged as a specialized strategy, targeting cytokine-mediated regulatory pathways to rectify immunological imbalances. Proinflammatory cytokines are key players in inducing and propagating autoimmune inflammation, highlighting the potential of cytokine therapies in managing autoimmune conditions. This review discusses the etiology of autoimmune diseases, current therapeutic approaches, and prospects for future drug design.

## 1. Introduction

Autoimmune disorders arise when the immune system erroneously attacks healthy cells, mistaking them for foreign invaders. This pathological misidentification leads to inflammation, tissue destruction, and impaired organ function. Under normal physiological conditions, the immune system adeptly identifies and eliminates potentially dangerous pathogens, such as bacteria, parasites, fungi, viruses, and even foreign tissues like tumors, without harming the body. However, the immune system can malfunction in two primary ways: first, through immune deficiency disorders, where one or more components of the immune system fail to protect the body from pathogens, and second, through autoimmune diseases [1]. The fundamental cause of autoimmune diseases lies in the immune system’s inability to distinguish self from non-self, a phenomenon often described as a breakdown of immunological tolerance. Understanding this breakdown is central to comprehending autoimmune disorders.

The critical importance of autoimmunity in clinical disease was first elucidated approximately 50 years ago by Macfarlane Burnett. His introduction of the “forbidden clone” theory, which earned him a Nobel Prize [2], marked a pivotal advancement in our understanding of autoimmunity, including lymphoid cell development, thymic education, apoptosis, and the elimination of autoreactive cells. Burnett’s work laid the foundation for subsequent research in the field [3].

The increasing prevalence of autoimmune diseases has prompted several theories, many of which may apply to multiple disorders. The development of autoimmune diseases is a complex process involving various factors, including genetics, environmental triggers, and immune dysregulation [4]. Genetic susceptibility plays a crucial role in the development of autoimmune diseases, as certain genes can increase the risk of these disorders [5]. The incidence of autoimmune illness among identical twins ranges from 12% to 67%, indicating that, in addition to environmental factors, stochastic or epigenetic processes are also relevant [6,7].

Environmental factors such as infections, diet, and exposure to toxins can trigger autoimmune responses in genetically susceptible individuals. The etiology of autoimmune diseases involves a complex interplay between genetic and environmental factors. For instance, individuals with particular genetic susceptibilities may have an increased risk of developing autoimmune disorders when encountering specific environmental triggers, including infections, toxins, or stressors [8]. The progress of autoimmune diseases has been determined in relation to the T helper 1(Th1)/Th2 cytokine balance, two T-cell subsets that cross-regulate each other [9,10]. In this context, Th1 mediates responses through cytokine production, such as interleukin 2 (IL-2), tumor necrosis factor (TNF)-α, and interferon (IFN)-γ, and through macrophage cytokines such as TNF-α, IL-1, IL-6, and IL-12. However, Th2 produces responses by IL-4, 1L-5, and IL-13 [9,11]. Cytokines are the main players in inflammatory responses in autoimmune conditions/diseases. Proinflammatory cytokines generally increase in autoimmune conditions and activate the cellular component of immunity at the site of inflammation. This primary inflammatory cascade helps along the progression of autoimmune pathology. The continuous activation of immune cells by cytokines can induce tissue damage and lengthen the autoimmune pathology. For example, in inflammatory bowel disease and psoriasis, IL-17 and IFN-γ produce inflammatory mediators and further matrix-degrading enzymes that cause tissue destruction and degradation. Similarly, fluctuations in cytokine levels can stimulate autoimmune flares, which are characterized by the worsening of symptoms and the intensification of disease severity. For example, a shift toward proinflammatory cytokines can magnify autoimmune responses and cause disease flares in conditions like systemic lupus erythematosus and vasculitis [12].

Autoimmune diseases have traditionally been categorized based on whether they target specific body tissues or cells, such as the receptors at the neuromuscular junction in myasthenia gravis or the beta cells of the pancreas in insulin-dependent diabetes mellitus [13]. Alternatively, they may impact multiple tissues, as seen in conditions like dermatomyositis/synthetase syndromes, which affect the skin, muscle, and lungs, or systemic lupus erythematosus, which involves the skin, kidneys, joints, bone marrow, and nervous system. Typically, the autoantigens targeted in tissue-specific autoimmune responses are localized to particular tissues, such as components of the acetylcholine receptor in myasthenia gravis or islet cell autoantigens in insulin-dependent diabetes mellitus [14]. Conversely, the autoantigens involved in systemic autoimmune processes are generally expressed in various cell types and tissues throughout the body [15].

In the past two decades, substantial research has advanced our understanding of the onset kinetics of autoimmune diseases. Harley and colleagues significantly contributed to this field by clarifying the timeline of autoantibody generation. They refuted the traditional notion that autoantibodies emerge only close to the onset of the disease. For instance, systemic lupus erythematosus (SLE) patients often possess autoantibodies long before the initial symptoms appear or the disease manifests [16]. Harley’s findings categorize antibodies in patients progressing toward an SLE diagnosis into two groups: (i) those detectable long before the first symptoms, such as anti-phospholipid antibodies (APL), anti-Ro antibodies, and antinuclear antibodies (ANAs), and (ii) those that markedly increase near the time of diagnosis, including anti-RNP, Anti-Sm, and, to a lesser extent, anti-DNA. This early presence of an immune response recognizing autoantigens has also been observed in other tissue-specific and systemic autoimmune diseases, such as type I diabetes and rheumatoid arthritis (RA) [17,18,19]. These crucial insights highlight that an immune response against autoantigens can lead to persistent tissue damage, with qualitative and/or quantitative changes in the autoimmune response contributing to the development of clinical symptoms [20].

Overall, the development of autoimmune diseases is a complex interplay between genetic, environmental, and immunological factors. Further research is needed to fully understand their underlying mechanisms [21].

In central tolerance (negative selection), immature T-cells (thymocytes) that strongly bind to self-antigens presented by thymic epithelial cells, dendritic cells, and macrophages undergo apoptosis. Conversely, peripheral tolerance prevents overly sensitive T and B cells from responding inappropriately to environmental stimuli, such as microbes and allergens [22]. All phases of autoimmune disease are associated with the breakdown of regulatory systems, which perpetuates chronic inflammation and ongoing immune responses. While some autoimmune conditions may enter a resolution phase, this phase is characterized by the partial and transient restoration of the balance between effector and regulatory responses. For assessing the risk of autoimmune diseases, a combination of genetic scores and autoantibody profiles can yield effective results, as seen in type 1 diabetes [23].

This review aims to provide a comprehensive overview of the mechanisms underlying autoimmunity, the breakdown of self-tolerance, predisposing genetic factors, and the current therapeutic strategies, with a focus on interleukin (IL)-related therapeutics. Recent advances in our understanding of these processes and the development of new treatment modalities underscore the importance of continued research in this field. Further elucidation of the underlying mechanisms is essential for the development of more effective therapies and the improvement of patient outcomes.

## 2. Mechanism of Autoimmunity

The effective functioning and maturation of the immune system depends on a complex network of signaling pathways. Disruptions in these pathways can lead to the proliferation and activation of self-reactive lymphocytes that target self-antigens, excessive cytokine production, and the release of autoantibodies. These events can result in the damage and destruction of normal tissue, causing autoimmune conditions.

Autoimmunity is triggered by a combination of genetic predisposition and environmental factors, reflecting the complexity of autoimmune diseases. Once initiated by these primary mechanisms, autoimmunity is further propagated by increasing inflammation and tissue damage, which can arise from various circumstances. Deficiencies in central or peripheral immunological tolerance may cause autoimmunity. Central tolerance, or negative selection, eliminates T or B cells reactive to the body’s own tissues, fostering self-tolerance. This occurs primarily in the thymus for T-cells and in the bone marrow for B cells, where cells that strongly bind to self-antigens are induced to undergo apoptosis [24]. In contrast, peripheral tolerance acts to prevent the over-reactivity of T and B cells that escape central tolerance, particularly for antigens not expressed in the thymus or bone marrow during central tolerance development. Mechanisms such as allergen deletion and suppression by regulatory T-cells (Tregs) are critical for maintaining peripheral tolerance [25]. The Th1/Th2 paradigm, introduced by Mosmann and Coffman, has significantly advanced our understanding of immune responses and autoimmune diseases by delineating the differentiation of T helper (Th) cells into Th1 or Th2 types based on their cytokine environment. This paradigm explains that Th1 responses are associated with acute-phase reactions to pathogens, whereas Th2 responses facilitate antigen elimination and disease recovery. It underscores the role of cytokines like interferon-gamma (IFNγ), produced by NK cells and Th1 cells, and IL-12 from dendritic cells in Th1 differentiation, while IL-4 is crucial for Th2 differentiation. These cytokines not only promote their own subset growth but also inhibit the development of the opposing subset, with Th1 cells enhancing immunoglobulin G2a synthesis and Th2 cells stimulating IgE and IgG1 production.

The broader context encompasses a variety of T-cell subsets, including cytotoxic T lymphocytes (CTLs), T helper (Th) cells, and regulatory T-cells (Tregs). Th cells are further subdivided into Th1, Th2, Th17, and Treg subsets, each characterized by distinct cytokine profiles and specific functions in immune regulation [26]. Th1 cells are known for producing cytokines such as IFNγ and IL-12, which are crucial for macrophage activation. In contrast, Th2 cells secrete cytokines including IL-4, IL-5, IL-10, and IL-13, which play essential roles in B-cell activation (Wang et al., 1999) [27]. The paradigm highlights how Th1 cytokines (e.g., IL-12, IFNγ, TNF-α, IL-1β) activate macrophages, enhancing their antigen-presenting capabilities, whereas Th2 cytokines (e.g., IL-4, IL-5, IL-10, IL-13) support B-cell activation and downregulate proinflammatory macrophage functions. The imbalance between Th1 and Th2 cells is directly linked to autoimmune pathologies, as observed in conditions like Guillain–Barré syndrome (GBS) and multiple sclerosis (MS), indicating a pivotal role in autoimmune disease mechanisms [28].

**Figure 1:**
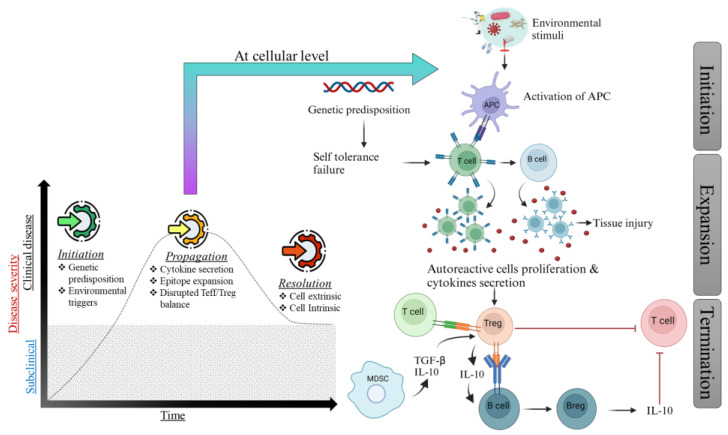
Autoimmune diseases progress through three major phases. In the initiation phase, a combination of environmental triggers and genetic predispositions leads to the breakdown of immune tolerance. This breakdown triggers the activation of autoreactive immune cells. During the amplification phase, these autoreactive cells escalate the immune response by releasing cytokine and attracting additional immune cells to the inflammation site. This phase can become self-perpetuating, often resulting in chronic inflammation. The resolution phase is characterized by the subsiding of autoimmune reactions. This occurs through the activation of cell-intrinsic inhibitory pathways and cell-extrinsic mechanisms, which collectively work to limit the effector response and restore the balance between T effector cells (Teffs) and T regulatory cells (Tregs). However, patients in this phase frequently experience disease relapses and remissions. These relapses, marked by a continuous struggle between pathogenic effector responses and regulatory mechanisms, can cause tissue damage, leading to the clinical manifestations of autoimmune diseases. It is important to note that the severity and duration of these phases can vary significantly, depending on the specific autoimmune disease and individual patient factors.

The gradual increase in the ratio of effector cells to regulatory cells is indicated by the proliferation of Teffs in the tissue and the decrease in or defective formation of Tregs [29]. Additionally, T helper type 1 (Th1) and Th17 cells secrete proinflammatory cytokines, including IL-17 and interferon-gamma (IFN-γ), promoting inflammatory responses and contributing to various autoimmune diseases, such as multiple sclerosis, collagen-induced arthritis, and rheumatoid arthritis (RA) [30]. Studies have demonstrated that the cytokine environment regulates Th1/Th17 cell responses and affects the production and function of Tregs. IL-23 promotes Treg plasticity by creating a population of Th17-like Tregs, which may play a significant role in autoimmune disease pathogenesis [31,32,33,34]. When the regulatory system that restricts effector responses is restored, the final phase, known as the resolution phase, is triggered, as evidenced by clinical remissions. Tregs are essential for modulating immune responses and mitigating inflammation. They secrete immunosuppressive cytokines, such as transforming growth factor-beta (TGF-β) and IL-10, which aid in repairing tissue damage caused by autoimmune activity. In cases where a resolution phase occurs, Tregs contribute significantly to this process [35]. Autoimmunity animal models have shown that Tregs are responsible for the resolution phase, as they are activated and stimulated by Teffs that produce IL-2 during the initiation and propagation phases [36]. Other cells also play a significant role in autoimmunity resolution. Regulatory B cells (Bregs) are involved in developing and activating T-cells, altering their phenotype to a regulatory one. Bregs also generate IL-10, reducing the production of inflammatory cytokines by dendritic cells (DCs) and inhibiting the development of Th17 and Th1 cells [37] (Figure 1).

## 3. Immune Tolerance

Immune tolerance is the immune system’s capability to recognize and differentiate the body’s own cells and molecules from foreign entities, thereby avoiding attacks by self-antigens. This mechanism is vital for maintaining bodily equilibrium and preventing the development of autoimmune disorders. Immune tolerance is essential for maintaining homeostasis by eliminating autoreactive T and B cells and by inactivating, suppressing, or deleting mature lymphocytes that escape central tolerance. Unlike the typical immune-mediated clearance of foreign antigens, immune tolerance is achieved through prior exposure to that antigen. Tolerance can be categorized into two types based on the site at which it is initially induced in the body: central tolerance, occurring in the thymus and bone marrow, and peripheral tolerance, occurring in other tissues and lymph nodes (peripheral tolerance) [38]. Although the processes by which central and peripheral tolerances are developed are diverse, their effects are comparable.

### 3.1. Central Tolerance

In the early 1960s, Jacques Miller and Max Cooper recognized T and B cells as defining elements of the antigen-specific adaptive immune system. T lymphocytes indirectly control immunological responses by providing soluble and membrane-bound signals that enhance the survival, growth, and differentiation of B cells, generating antibodies to support humoral immunity [39].

The T-cell receptor (TCR) recognizes peptide antigens presented by major histocompatibility complex (MHC) molecules on the surface of antigen-presenting cells. The interaction between the TCR and peptide–MHC complex is specific and highly sensitive, allowing T-cells to detect and respond to a wide range of foreign antigens [40]. T-cells recognizing self-antigens with high affinity are eliminated during their development in the thymus through a process called negative selection, which is critical for preventing autoimmune responses. In contrast, regulatory T-cells (Tregs) recognize self-antigens and prevent autoimmunity by suppressing the activity of self-reactive T-cells [41]. The alpha (α) and beta (β) chains in the TCR complex are produced through somatic recombination, which is critical for the specificity of T-cell antigen recognition. These chains are formed by the somatic recombination of various genetic elements, involving the insertion of a few nucleotides at the recombination site [42]. This combinatorial process generates a vast TCR repertoire with more than 10^10 unique receptor combinations and is a critical factor in the capacity of the T-cell population to respond to new antigens. However, this repertoire is limited and skewed in each individual because each T-cell is selected by its capacity to bind a self-peptide in the context of the polymorphic and polygenic components of its ligand [43]. However, this repertoire is limited and skewed in each individual because each Tcell is selected by its capacity to bind a self-peptide in the context of the polymorphic and polygenic components of its ligand [44]. These polygenic components are molecules encoded by the MHC. This step of selection, known as positive selection, ensures that T-cells can recognize a foreign peptide antigen when they bind to proteins encoded by specific self-MHC alleles. These self-MHC alleles are typically MHC class I molecules for cluster differentiation 8+ (CD8+) T-cells and MHC class II molecules for CD4+ T-cells [45].

After the first selection, known as “positive selection”, T-cells undergo a second selection step called “negative selection”. This filtering phase is designed to eliminate T-cells with a significant binding affinity to self-peptides coupled to the same MHC molecules, thereby preventing autoimmunity. These autoreactive T-cells are produced by chance during the stochastic gene rearrangement process responsible for generating the TCR. The interaction between developing T-cells, medullary thymic epithelial cells (mTECs), and dendritic cells (DCs) from bone marrow is necessary for the negative selection process [46].

The mTECs express a transcriptional activator known as the autoimmune regulator (AIRE), which facilitates the expression of numerous tissue-specific antigens. This antigen presentation by mTECs to developing T-cells is crucial for the elimination of self-reactive CD4+ and CD8+ T-cells, ensuring central tolerance [47,48]. The clinical presentation of autoimmune polyglandular syndrome type 1 (APS-1), a severe autoimmune condition involving multiple organs in individuals with a mutated AIRE gene, underscores the crucial role played by AIRE-positive mTECs in maintaining central tolerance [49].

### 3.2. Peripheral Tolerance

Although the thymus efficiently eliminates self-reactive cells, numerous self-reactive T-cells can still evade the thymic negative selection. As a result, peripheral mechanisms are necessary to maintain self-tolerance. Peripheral tolerance is regulated by various cell types and activities, including those of the adaptive and innate immune systems and signaling components inside antigen-presenting and T-cells [50,51].

The protein CD28, the first T-cell costimulatory receptor discovered, is found on the surface of most T-cells. CD80 and CD86 are two ligands predominantly expressed on antigen-presenting cells. Only antigen-presenting cells that detect nominal antigens, referred to as “signal one”, respond to a second costimulatory signal, which is essential for the full activation of T-cells [52].

Despite the importance of CD28 for lymphocyte activation and survival, some antigen-experienced T-cells lose CD28, resulting in reactivation without CD28 involvement. These CD28-negative T-cells are typically categorized as terminally differentiated, are antigen-specific, and are referred to as memory T-cells (TMs) [53]. The loss of CD28 is becoming increasingly significant in the context of organ transplantation, particularly with costimulation blockade therapies. Evidence suggests that CD28-negative T-cells are involved in chronic viral infections such as HIV, hepatitis C virus, and human parvovirus B19. Additionally, a heterogeneous CD8+CD28- T-cell phenotype has been detected in autoimmune diseases. The loss of CD28 is also associated with type 1 diabetes, multiple sclerosis, Graves’ disease, ankylosing spondylitis, and rheumatoid arthritis [54].

Inhibiting costimulatory pathways leads to antigen-specific cell apoptosis, clonal population inactivation, and tolerance induction. In autoimmune diseases or organ transplantation, costimulatory inhibition using monoclonal antibodies, soluble versions of the high-affinity receptors CD80 and CD86, and cytotoxic T-lymphocyte-associated protein 4 (CTLA-4) promotes tolerance in animal models [55]. The two-signal paradigm of T-cell activation has been confirmed and expanded by discovering additional costimulatory pathways. Examples of these pathways include CD154-CD40, CD11A-CD54, CD18-CD54, and CD2-CD58. Novel therapeutic alternatives are available for inhibiting the activity of autoreactive T-cells and generating long-term tolerance without continuous treatment [56]. The regulation of T-cell activation involves costimulatory pathways and negative regulators or checkpoints on the surface of activated T-cells. In contrast to costimulatory pathways, these checkpoints, such as CTLA-4 and programmed death 1 (PD-1), inhibit immune activity when activated by their respective ligands, resulting in the induction of active tolerance [57,58].

The deliberate blockade of checkpoint pathways provided direct evidence for their crucial role in the development of immune tolerance. Gene deletion studies or therapies involving checkpoint inhibitors have demonstrated that such interventions can exacerbate autoimmune conditions and, in some cases, dismantle the tolerance established by previous treatments [59]. Consequently, targeting checkpoint pathways has led to the development of a novel class of medications capable of modulating immune tolerance. The inhibition of these pathways has revolutionized immunotherapy, transforming previously incurable diseases into manageable conditions. Notable examples include lupus [60], melanoma [61], and non-small-cell lung cancer [62]. In the subsequent section, we will explore various checkpoint agonists used in the treatment of autoimmune diseases and organ transplant rejection [58].

In the context of inhibiting costimulatory pathways, monoclonal antibodies such as abatacept (CTLA-4-Ig) and its derivative belatacept have been utilized to interfere with CD28-mediated costimulation. This inhibition results in reduced T-cell activation and prolonged graft survival in transplantation models [63]. Moreover, soluble CTLA-4-Ig fusion proteins bind to CD80 and CD86, thereby preventing their interaction with CD28. This blockade interrupts the crucial second signal required for T-cell activation. These therapeutic strategies highlight the potential of targeting costimulatory pathways in clinical applications. Nonetheless, extensive research and clinical trials are imperative to thoroughly assess their efficacy and safety in human subjects [64].

Specific cell populations play a crucial role in reducing pathogenic immune responses that erroneously target self-tissue, thus contributing to the development of peripheral tolerance. These populations include naturally occurring regulatory T (Treg) cells, in vitro-induced Treg cells, IL-10-producing type 1 regulatory T (Tr1) cells, and TGF-β-producing type 3 helper T (Th3) cells [65]. Among these subsets, Tregs, a subset of self-reactive CD4+ T-cells, emerge due to the expression of a master transcriptional repressor called forkhead box P3 (FOXP3). The differentiation of mature CD4+ T-cells into the Treg fraction is essential for maintaining immunological homeostasis and can be influenced by FOXP3 expression. The disruption of FOXP3 function, either genetically or pharmacologically, leads to severe autoimmune diseases that result in early mortality unless the patient receives a bone marrow transplant [66].

Tregs are generated through an alternative developmental process in the thymus. A subset of developing T-cells expressing TCRs with high affinity differentiate into Treg precursors that upregulate FOXP3, resulting in a stable epigenetic state and a mature self-reactive population of thymus-derived Tregs (tTregs) that populate lymphoid and non-lymphoid tissues in the periphery during negative selection [67]. The peripheral function of Tregs is to inhibit self-reactivity and promote tissue repair and regeneration. Tissue-specific antigen presentation by medullary thymic epithelial cells (mTECs) expressing AIRE contributes to the formation of tTregs. Furthermore, AIRE expression in a fraction of bone marrow-derived cells in peripheral lymphoid organs impacts tolerance in peripheral tissues, illustrating the relationship between peripheral tolerance and thymic T-cell development [68].

Tregs can also be produced in the immune periphery when naive CD4+ T-cells encounter antigens in the presence of suppressive factors such as TGF-β, IL-10, bacterial metabolic products, or altered stimulatory pathways. These conditions facilitate the immune system’s exposure to antigens alongside suppressive factors. Under certain circumstances, antigen presentation may lead to the persistent expression of FOXP3 and the transition of conventional T-cells into peripherally derived regulatory Tregs (pTregs) [69]. Therefore, unlike tTregs, which originate from T-cells during negative selection in the thymus due to high self-reactivity, pTregs originate from a traditional peripheral T-cell repertoire selected for low self-reactivity [70]. This recognition process expands Tregs, enhancing their effectiveness in suppressing inflammatory responses. Additionally, pTregs can detect altered proteins such as citrullinated and hybrid peptides and phosphorylated proteins, often present in autoimmune conditions but not in the thymus. The combination of tTregs and pTregs, along with IL-10-generating Tr1 cells and TGF-β-producing Th3 cells, may offer the broadest antigen-recognition repertoire for managing pathogenic self-reactivity. These cell types work synergistically to maintain tolerance. Furthermore, because thymic involution occurs throughout maturation, the establishment and maintenance of tolerance may vary with age, with peripheral pathways playing a more critical regulatory role in autoimmunity in adults than in children [71].

Antigen-presenting cells (APCs), including tolerogenic dendritic cells (DCs), myeloid lineage suppressor APCs, immature macrophages, and specific B-cell subsets, collaborate with Treg cells and other suppressor T-cells to regulate the immune system. These cells arise in response to various cell-surface and soluble stimuli, some of which are modulated by Tregs [72,73,74]. For example, Tregs express high levels of the checkpoint protein CTLA-4, which can inhibit CD28-mediated costimulation and send inhibitory signals that transform APCs into tolerogenic cells. Tregs and other regulatory cells produce cytokines such as IL-10, IL-35, and TGF-β, as well as other soluble molecules involved in metabolism, such as indoleamine 2,3-dioxygenase. These cytokines suppress the immune system by inhibiting T-cell activity. Additionally, Tregs can induce the production of myeloid-derived suppressor cells and modify antigen presentation [75].

**Figure 2:**
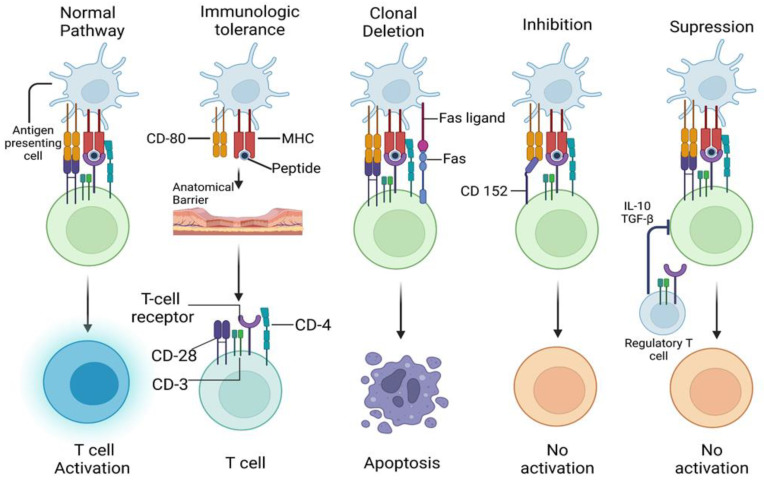
Peripheral mechanisms of T-cell tolerance induction. This figure illustrates various methods by which T-cell tolerance is induced peripherally. It includes immunologic ignorance, where T- cells physically separated from their specific antigen (e.g., by the blood–brain barrier) cannot become activated. T-cells expressing the Fas (CD95) molecule undergo apoptosis upon interaction with cells expressing Fas ligand, a process known as deletion. Inhibition of T-cell activation is exemplified by the binding of CD152 to CD80 on antigen-presenting cells. Additionally, regulatory T-cells suppress other T-cells, likely through the production of inhibitory cytokines such as interleukin-10 and transforming growth factor β (TGF-β).

Tregs also express CD39 and CD73, which alter the duration, intensity, and chemical nature of purinergic signals transmitted to immune cells through the conversion of adenosine diphosphate (ADP) or adenosine triphosphate (ATP) to adenosine. Consequently, metabolic byproducts produced by the gut microbiome, such as short-chain fatty acids like butyrate, can significantly influence immune system functioning [76]. These regulatory factors can act on APCs to stimulate the generation of Tr1 cells and Tregs and can directly affect pathogenic T-cells by altering their differentiation, trafficking, and functions. Studies of oral tolerance with short-chain fatty acids support a robust relationship between the immune system and the microbiota [77] (Figure 2). Autoimmune lymphoproliferative syndrome (ALPS) and ALPS-like syndromes are conditions characterized by defects in the FAS-mediated apoptosis pathway, leading to an imbalance of lymphocytes and autoimmune manifestations. ALPS is marked by excessive lymphocyte proliferation, cytopenia, and organ-specific autoimmunity. ALPS-like syndromes exhibit similar clinical features due to genetic mutations affecting components of the apoptosis pathway. These syndromes highlight the critical role of apoptosis in maintaining immune homeostasis and preventing autoimmunity [78].

Consequently, a dynamic, homeostatic immune system has developed in response to internal and external aggressions. As seen in patients with coronavirus disease 2019 (COVID-19) and autoimmunity, there is a fine line between the need for strong Teffs to fight against external pathogens and the need for homeostatic modulation of the immune system to stop unwanted autoinflammation [79].

## 4. Breakdown of Tolerance

The thymus plays a crucial role in T-cell tolerance development because it is where T-cells mature and undergo positive and negative selection to ensure they recognize foreign antigens and do not attack self-antigens. The thymus selects T-cells through clonal deletion, eliminating cells that react strongly to self-antigens. However, not all self-reactive T-cells are eliminated; some escape into the periphery [80]. For example, the INS gene, which modulates susceptibility to autoimmune diabetes, regulates insulin expression levels in the thymus. Genetic variation in this gene can lead to altered thymic insulin expression, potentially disrupting the negative selection process. This disruption may allow self-reactive T-cells to evade central tolerance and migrate to the periphery, contributing to the onset of autoimmune diabetes [81]. The activation of potentially self-reactive T-cells in the normal repertoire by pathogenic microorganisms is another mechanism that may trigger autoimmune diseases. Infections contribute to the development of autoimmune illnesses, including multiple sclerosis and type 1 diabetes [82,83]. The release of sequestered autoantigens due to tissue damage also leads to autoimmunity following an infection. Other mechanisms related to the development of autoimmunity include the activation of a large fraction of the T-cell population by superantigens and the induction of inflammatory cytokines and costimulatory molecules by microbial products. This form of bystander activation has been shown to induce diabetes in mice with an autoimmune etiology [84].

Molecular mimicry is a possible mechanism for activating autoreactive T-cells, where a structural resemblance exists between self-antigens and microbial antigens [85]. Some T-cells recognize both microbial and self-peptides that share amino acid sequences. For example, molecular mimicry between multiple factors are likely to contribute to the onset of autoimmune diseases. It is important to note that a single T-cell receptor can recognize multiple peptides, some of which may not have significant sequence homology [86]. The theory that cross-reactivity between microbial and self-peptides is solely responsible for autoimmunity may be oversimplified, as such cross-reactivity could also trigger protective immune responses. Interestingly, a strong correlation exists between a high number of infections during the first year of life and a reduced risk of developing autoimmune diabetes [87].

There are additional checkpoints that prevent autoimmunity. For instance, patients with multiple sclerosis (MS) often fully recover from optic neuritis, a common early symptom of the disease. In individuals who have experienced a single episode of optic neuritis and those who later develop MS, T lymphocytes can recognize central nervous system antigens [88,89].

## 5. Predisposing Factors of Autoimmune Diseases

Autoimmune disorders are conditions with an incidence in the general population estimated between 3 and 5% [90]. Despite significant advances in detecting and treating autoimmune diseases, the etiological processes leading to clinical pathology remain inadequately understood. Researchers continue to investigate the specific origins of over 80 autoimmune diseases. Several risk factors can alter immunological tolerance and contribute to the development of autoimmune disorders. While these factors may increase susceptibility to autoimmunity, they do not necessarily guarantee disease onset. Some common predisposing factors of autoimmune diseases are discussed below.

### 5.1. Gender

Many autoimmune disorders exhibit significant sex dimorphism, with women being more susceptible than men. Gonadal hormones and direct effects of the X chromosome are the primary contributors to this dimorphism. For instance, the gender ratio in systemic lupus erythematosus (SLE) shifts from 2:1 before puberty to 9:1 after puberty, highlighting the role of estrogen in SLE susceptibility [91]. In contrast, androgens tend to reduce immunological responses and autoimmunity. These findings suggest a substantial involvement of sex hormones in the immune system’s functioning. Multiple genes producing immunologically important molecules, such as inflammatory cytokines and Toll-like receptor (TLR) signaling molecules, are widely regulated by female hormones [92,93]. Estrogens can interfere with B-cell tolerance, while T-cell tolerance may also be affected because estrogen downregulates and androgens upregulate AIRE expression in the thymic epithelium [94,95]. Furthermore, sex hormones and the microbiome influence each other, and sex differences in microbiota composition may contribute to sex biases in androgen-induced regulation of autoimmunity. The interaction between sex hormones and the microbiome can modulate immune responses and potentially impact the development and progression of autoimmune diseases.

### 5.2. Environment

Identifying environmental factors is crucial for understanding individual vulnerability to autoimmune diseases. However, relatively few agents have a clear role in immunological responses, making general risk factor identification challenging. Environmental factors include diet, microbiota, infections, and xenobiotics such as cigarette smoke, pharmacological agents, hormones, ultraviolet (UV) radiation, silica, solvents, heavy metals, vaccinations, and collagen or silicone implants [96,97]. Other environmental triggers, such as sunlight in lupus erythematosus, may work similarly to infections by causing tissue damage or altering a host molecule to the point that it becomes immunogenic, as seen in chemically or drug-induced autoimmune disorders. In addition, a permissive genetic background is also required [98].

Autoimmunity can arise within the body if an intracellular self-molecule is produced abnormally and presented on the cell surface. The “internal” environment is responsible for the paraneoplastic autoimmune associations with ovarian, lung, and breast cancers, where an antigen associated with the tumor elicits remarkable autoimmune responses, damaging structures such as cerebellar, motor, or sensory neurons; nerve terminals, as in Lambert–Eaton myasthenic syndrome; or retinal cells [99]. These disorders represent a mistaken immunological defense against cancer, often preceding the overt manifestation of tumors and possibly inhibiting tumor spread [100].

The internal environment is also indirectly significant because hormones influence the female predisposition to autoimmunity, with autoimmune thyroid disease and type 1 diabetes potentially manifesting during the postpartum period. The purported consequences of psychological stress acting via neuroendocrine pathways are not well defined [101].

### 5.3. Genetic Risk Factors

Autoimmune diseases have a complex etiology, involving both genetic and environmental factors. Extensive research has identified various genes associated with susceptibility to autoimmune diseases. These genes can influence the immune response to specific autoantigens, the specificity of antigen receptors on T and B lymphocytes, or the sensitivity of target tissues to autoimmune attacks. Additionally, genes affecting tolerance, apoptosis, or inflammatory responses contribute to general susceptibility to autoimmunity [72].

One of the most well-known genetic factors associated with autoimmune diseases is the major histocompatibility complex (MHC). Located on chromosome 6, the MHC plays a critical role in the immune system’s recognition of self- and non-self-molecules. Specific MHC alleles are linked to increased susceptibility to autoimmune diseases, while others may provide immune protection. For instance, the human leukocyte antigen-DRB1 (HLA-DRB1) gene is strongly associated with susceptibility to rheumatoid arthritis (RA), and certain HLA-DQ and HLA-DR alleles offer protection against type 1 diabetes [102]. Other genes associated with autoimmune disease susceptibility include those involved in immune cell signaling and activation, such as protein tyrosine phosphatase non-receptor type 22 (PTPN22), CTLA-4, and CD40. Variants of these genes can alter the threshold for immune cell activation, increasing the risk of autoimmunity. Additionally, genes involved in immune regulation and tolerance, such as FOXP3, IL2RA, and TNF receptor superfamily member 25, have also been linked to autoimmune disease susceptibility [103].

Autoimmune diseases are prevalent, with the majority of cases arising from a combination of environmental factors and polygenic genetic predispositions. Inborn errors of immunity (IEIs) are a variety of genetic disorders that represent a diverse group of genetic disorders, encompassing a group of nearly 500 inherited disorders that compromise immune system functionality, in which parts of the human immune system are missing or dysfunctional, leading to increased susceptibility to infections, autoinflammatory conditions, and autoimmune diseases [1,104]. The term “primary immunodeficiencies” has recently been replaced with “inborn errors of immunity” (IEIs) to address the wide range of clinical conditions, which cover not only immune deficiencies but also excessive or defective immune responses [105]. IEIs caused by abnormalities in innate signaling pathways, complement proteins, or phagocytic cells are categorized as innate immune defects. Complement protein defects initiate bacterial infections related to autoimmunity, while phagocytic component defects lead to fungal and bacterial infections and dysregulate the immune system [106,107]. If uncontrolled inflammatory cytokines are produced, then they will cause autoinflammatory syndromes characterized by repetitive fevers and organ system inflammation [108].

The adaptive immune response is characterized by its specificity and the creation of immunological memory via T-cells and B cells. T-cells are crucial in eradicating cancerous or virally infected cells, activating other immune cells, such as B cells and macrophages, and preventing immune dysregulation. The term “combined” immune deficiency refers to defects in T-cells that ultimately lead to abnormalities in B cells [109]. Common variable immunodeficiency (CVID) is a type of inborn error of immunity (IEI) characterized by reduced serum immunoglobulin levels and impaired specific antibody production [110]. For instance, familial hemophagocytic lymphohistiocytosis (fHLH) involves the impaired regulation and activity of cytotoxic cells, resulting in uncontrolled immune activation and potentially life-threatening cytokine storms. fHLH7 typically manifests in childhood, while secondary forms occur in adults, often triggered by autoimmune diseases, lymphomas, or certain viral infections. This hyperinflammatory state is marked by excessive immune responses. Adults with inborn errors of immunity (IEIs) often encounter significant delays in both diagnosis and treatment [111,112].

Autoimmune poly-endocrinopathy candidiasis ecto-dermal dystrophy (APECED) is a monogenic autoimmune disease caused by mutations in the AIRE gene that dysregulate central immune tolerance and induce the escape of self-reactive T lymphocytes, which invade numerous endocrine (e.g., adrenals, parathyroids, gonads, pancreas, thyroid) and non-endocrine (e.g., skin, kidneys, liver, slivery glands, spleen, enamel, stomach, small intestine, lungs) organs and give rise to tissue destruction [113]. APS-1 generates a remarkable reaction with both interferon-omega (IFN-Ω) and interferon-alpha (IFN-α). It is also noteworthy that there may be a reaction against interleukin 22 (IL-22). This culminates in damage to endocrine organs, commonly manifesting as hypercalcemia and nephrocalcinosis and the dysregulation of pituitary hormones, such as growth hormone deficiency [114]. Although central tolerance mechanisms primarily contribute to the pathogenesis of APECED, various peripheral mechanisms also play a crucial role in regulating the immune system. These peripheral components are involved in maintaining homeostasis and peripheral tolerance by controlling residual autoreactive clones that escape negative selection in the thymus. These mechanisms are essential in reducing or halting reactivity to self-antigens, thereby preventing autoimmune responses [115].

The life expectancy of patients with APECED is dependent upon the severity of the disease. The most life-threatening complications include severe malabsorption, fulminant necrotizing hepatitis, and tubulointerstitial nephritis. Inadequate hormonal replacement therapy or suboptimal management of Addisonian crises can significantly elevate the risk of mortality. Furthermore, patients with persistent oral candidiasis are at a heightened risk of developing esophageal squamous cell carcinoma. Understanding the molecular mechanisms and genetic foundations of IEIs is crucial for accurate diagnosis, prognosis, and the development of targeted therapeutic strategies for managing autoimmune diseases [116].

Despite the significant progress in identifying genetic factors associated with autoimmune diseases, the genetic basis of many autoimmune diseases remains poorly understood. Understanding the genetic basis of autoimmunity is relevant for identifying individuals at risk and developing targeted treatments based on the specific genetic profile of the patient [117]. Genome-wide association studies (GWASs) have been used to identify common genetic variants associated with several autoimmune diseases, including RA, systemic lupus erythematosus, and type 1 diabetes [118]. These studies have identified hundreds of genetic variants, many in non-coding regions of the genome, suggesting they may impact gene regulation or splicing [119,120]. GWASs has highlighted the complexity of the genetic architecture of autoimmune diseases. Many identified variants have small effect sizes, and most of the heritability of autoimmune diseases remains unexplained. Additionally, there is significant overlap in genetic risk factors between different autoimmune diseases, suggesting shared genetic pathways [121].

More advanced genetic technologies, such as whole-genome sequencing and single-cell sequencing, are being used to address these challenges. These approaches can identify rare variants with large effect sizes and genetic variants that impact gene expression or protein function [122]. Furthermore, they help unravel the cellular and molecular pathways underlying autoimmune diseases, providing new targets for therapeutic intervention.

### 5.4. Infections

The relationship between infectious agents and autoimmune diseases has been extensively studied through epidemiological research, laboratory investigations, and experimental models. Various viruses, bacteria, fungi, and parasites have been implicated in the initiation or promotion of autoimmune disorders, such as rheumatoid arthritis (RA), thyroid disorders, primary biliary cirrhosis, type 1 diabetes, and autoimmune hepatitis [96]. For instance, a case study linked hepatitis A virus infection to the onset of severe type 1 diabetes in a 38-year-old man who developed diabetes following acute hepatitis A [123]. A well-known example of the connection between infection and autoimmunity is acute rheumatic fever, which results from Streptococcus pyogenes exposure in genetically susceptible individuals. Molecular mimicry between the bacterial M protein and human lysoganglioside triggers autoimmunity in acute rheumatic fever [124].

The term “molecular mimicry” was coined by Damian in 1964, who suggested that some microbial antigenic determinants resemble host epitopes, potentially inducing an autoimmune response [125]. Numerous examples of molecular mimicry have been documented, though its therapeutic relevance can sometimes be unclear. Epidemiological and clinical studies, along with experimental data, strongly support the role of enteroviruses, particularly coxsackievirus B (CVB), in the pathogenesis of type 1 diabetes mellitus (T1DM). Enteroviral infections can trigger immune responses that lead to the destruction of pancreatic β-cells, demonstrating the interplay between genetic susceptibility and environmental factors in autoimmunity. CVB infections can induce mechanisms such as molecular mimicry and bystander activation, leading to β-cell destruction [126]. Epstein–Barr virus (EBV) infection is associated with triggering autoimmune responses targeting the central nervous system. This viral-mediated autoimmunity involves mechanisms such as molecular mimicry and the activation of autoreactive T-cells, contributing to the demyelination and neurodegeneration characteristic of multiple sclerosis (MS). The interaction between genetic susceptibility and EBV infection underscores the complex etiology of autoimmune diseases like MS [127].

A single T-cell receptor can recognize several peptides, not all with robust sequence homology. Thus, the hypothesis that cross-reactivity between microbial and body peptides alone explains autoimmunity onset is overly simplistic. However, there is a strong correlation between a high number of infections in the first year of life and a significantly lower risk of developing autoimmune diabetes [128]. In 1989, David P. Strachan proposed the “hygiene hypothesis” of allergic disease. This hypothesis posits that decreased exposure to infectious agents, microorganisms, and parasites in early childhood due to modern sanitation practices leads to an under-stimulated immune system. This reduced microbial exposure adversely affects immune system development, increasing susceptibility to allergic diseases and autoimmune disorders. This hypothesis helps explain the rising prevalence of allergies and autoimmune diseases in developed countries [129].

Infectious pathogens induce autoimmunity through several mechanisms, including molecular mimicry, epitope spread, bystander activation and stimulation of pattern recognition receptors, viral persistence, and autoinflammatory activation of innate immunity.

### 5.5. The Gut Microbiota

There is ample evidence that commensal bacteria in the gastrointestinal system, known as the gut microbiota, influence the development of autoimmunity [130]. Studies on newborns reinforce this argument. For instance, a study on the microbiomes of 33 HLA-matched infants, where monthly samples were collected from birth to 3 years of age, exemplifies this theory. The study participants were selected based on the presence of HLA risk alleles associated with type 1 diabetes mellitus [131].

During the trial, 11 out of 33 babies seroconverted to serum autoantibody-positive, and four developed type 1 diabetes. The microbiome taxonomy varied significantly within the same individual. Those who acquired autoantibodies had specific alterations in their microbiomes, including increased pathobionts and reduced bacteria that produce short-chain fatty acids [132].

Several factors influence gut microbiota, and there is a correlation between gastrointestinal motility and the use of pharmaceutical drugs, including antibiotics, nonsteroidal anti-inflammatory drugs, tobacco, and alcohol. These factors can trigger improper signaling along the gut–brain axis, impacting central nervous system function and leading to illness [133]. The gut microbiome interacts with the host, other species, and environmental variables. Exogenous viruses and the virome—the genomes of all viruses inhabiting a host—interact with the gut microbiota and reside in commensal bacteria [134,135]. Additionally, the microbiota and immune systems interact closely, with malnutrition affecting both the innate and adaptive immune systems and the microbiota. The microbiota functions as a barrier against enteropathogenic infections and can be compromised by starvation or immune system dysfunction [136]. Changes in the microbiota are associated with autoimmune diseases, affecting the gastrointestinal mucosa, which is in close contact with luminal contents. These diseases include celiac disease and autoimmunity directed against distant locations, such as type 1 diabetes and RA [137,138,139].

### 5.6. UV Radiation

UV radiation is a well-known environmental factor that triggers various immune responses. While it is necessary for vitamin D production and has therapeutic applications for some autoimmune skin disorders, such as psoriasis and vitiligo, excessive exposure to UV radiation has been linked to the development of autoimmune diseases. For example, UV radiation is associated with systemic lupus erythematosus (SLE), an autoimmune disease affecting multiple organs and tissues. UV radiation causes DNA damage and triggers the release of self-antigens, which stimulates the immune system and leads to autoimmunity. Additionally, UV radiation disrupts immune function by altering the behavior of immune cells and cytokine production [140].

UV radiation can also exacerbate existing autoimmune diseases. For instance, UV exposure worsens symptoms in patients with SLE, leading to increased skin rashes, arthritis, and kidney involvement. Similarly, UV radiation is linked to the exacerbation of symptoms in patients with rheumatoid arthritis (RA). Moreover, UV radiation interferes with the effectiveness of immunosuppressive medications used to treat autoimmune diseases, complicating the management of these conditions. Given the potential impact of UV radiation on autoimmune diseases, it is important to take appropriate measures to limit exposure, such as wearing protective clothing, applying sunscreen, avoiding midday sun exposure, and using artificial light sources that do not emit UV radiation [141].

### 5.7. Vitamin D Deficiency

1,25-Dihydroxyvitamin D (1,25 OH2 vit D), a steroid hormone synthesized from vitamin D, plays a crucial role in regulating blood calcium and phosphorus levels. Various human organs and cells express the vitamin D receptor and 1-hydroxylase, increasing interest in the potential extra-skeletal effects of vitamin D [142]. Vitamin D is essential in preventing and managing various acute and chronic conditions, including autoimmune diseases [143,144]. Furthermore, genetic studies have demonstrated an association between susceptibility to thyroid autoimmunity and gene polymorphisms of several proteins and enzymes involved in vitamin D function and the vitamin D receptor [145].

The association between vitamin D and autoimmune diseases is substantiated by epidemiological research, which indicates a higher prevalence of these diseases in regions with limited sunlight exposure. This correlation suggests that decreased synthesis of vitamin D may contribute to the increased incidence of autoimmune conditions [146].

### 5.8. Alcohol

Alcohol consumption has been linked to systemic inflammation and the exacerbation of various chronic health conditions, raising concerns about its potential role as a risk factor for autoimmune diseases. Interestingly, recent human and animal studies suggest that alcohol may have a protective effect against certain autoimmune disorders [147]. While excessive alcohol consumption is a known risk factor for psoriasis, moderate alcohol intake may reduce the risk of developing some autoimmune diseases [148]. Alcohol consumption can induce autoimmunity in the liver by disrupting complex cellular signaling pathways involving hepatic parenchymal and non-parenchymal cells, as well as immune cells. Chronic alcohol use sensitizes Kupffer cells, the liver macrophages, to endotoxins via the TLR-4 pathway, leading to inflammation and hepatocellular necrosis. The hyper-reactivity of Kupffer cells to endotoxins triggers the production of cytokines and TNF, resulting in liver inflammation and necrosis [149]. Therefore, while moderate alcohol consumption might reduce the risk of certain autoimmune diseases, chronic and excessive alcohol intake has detrimental effects on liver health and can contribute to the development of autoimmune disorders.

### 5.9. Drugs

Drug allergy refers to a specific and consistent immune response to a medication, often presenting with skin symptoms and eruptions. In clinical practice, it is common to encounter patients with autoimmune disorders who also have medication allergies. Individuals with Sjogren’s syndrome (SS), systemic lupus erythematosus (SLE), and adult-onset Still’s disease (AOSD) exhibit a high incidence of drug allergies [150].

Due to limited research in this area, the detailed symptoms, true frequencies, and underlying mechanisms of drug allergies in autoimmune patients remain unclear. However, the occurrence of autoimmune diseases following severe drug eruptions suggests a possible causal relationship between these conditions [151]. SLE, one of the most prevalent autoimmune disorders, affects approximately 10% of the global population. The first documented case of drug-induced lupus erythematosus (DILE) was in 1945, with sulfadiazine identified as the causative agent. Since then, over 90 drugs across more than 10 pharmacological classes have been linked to lupus. The incidence of DILE is estimated to range between 15,000 and 20,000 cases annually, accounting for 10% of SLE patients. Risk factors for DILE include slow acetylation, certain serologic characteristics such as HLA-DR4 and HLA-DR0301, a complement C4 null allele, and being female [152].

## 6. Personal Care Products and Cosmetics

**Figure 3:**
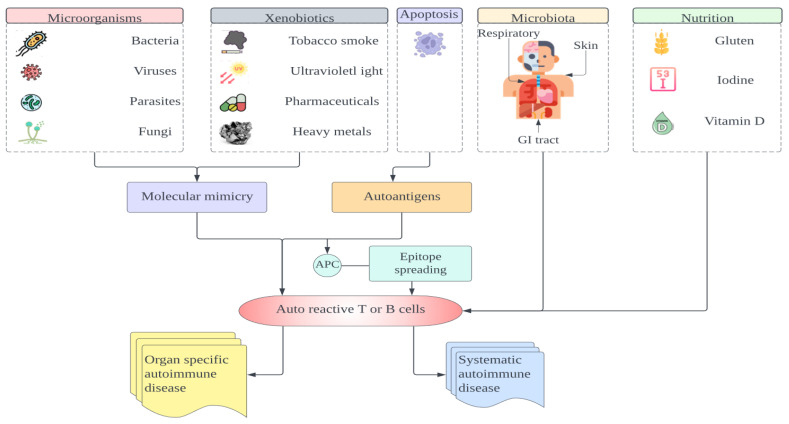
Environmental factors and their role in autoimmunity.

Personal care products, such as shampoos, hair dyes, and cosmetics, contain various natural and synthetic chemicals that may trigger autoimmunity. Some components, like acrylamides, are similar to those implicated in drug-induced lupus. For instance, nail polish has been identified as a risk factor for primary biliary cirrhosis and systemic lupus erythematosus (SLE) [153]. In a Canadian study by Cooper et al. (2010) involving 258 SLE cases and 263 controls matched for sex, age, and location, individuals who worked with paints, dyes, or film development had strong associations with those who used nail polish or received nail treatments. Lipsticks contain substances such as eosin, phthalate, and 2-octynoic acid, which are linked to autoimmune disorders. Eosin, a red dye, has been associated with photosensitivity and lupus flares. In experimental settings, phthalate has been shown to induce anti-DNA antibody responses and SLE-like disease. 2-Octynoic acid, a xenobiotic, can induce anti-mitochondrial antibody responses in primary biliary cirrhosis (PBC) by modifying the immunodominant E2 component of the pyruvate dehydrogenase complex [136]. Additionally, a significant group of electrophilic drugs, including commonly used nonsteroidal anti-inflammatory drugs and acetaminophen, may contribute to mimicry and loss of tolerance to the E2 component of the mitochondrial pyruvate dehydrogenase complex caused by xenobiotics [154] (Figure 3).

### Smoking, Silica, Tropospheric Pollutants, and Solvents/Pesticides

Exposure to various substances, including silica, cigarette smoke, tropospheric pollutants, and industrial solvents, has been associated with the development of autoimmune disorders [155,156]. The specific mechanisms through which these chemicals induce autoimmunity remain unclear and appear to vary based on the nature of the interaction and the type of exposure. Systemic lupus erythematosus (SLE) serves as a model for understanding how environmental factors can transform normal antigen-specific B helper T-cells into autoreactive, cytotoxic, and proinflammatory T-cells. This transformation leads to lupus-like conditions in animal models and similar pathological changes in humans [157]. Increasing evidence suggests that exposure to industrial solvents is linked to the onset of autoimmunity. These substances may act as triggers that initiate or exacerbate autoimmune responses [158,159].

Cigarette smoking has been identified as a significant risk factor for several autoimmune diseases, including rheumatoid arthritis (RA) and systemic lupus erythematosus (SLE). The mechanisms by which smoking induces autoimmunity include the increased production of proinflammatory cytokines, oxidative stress, and the formation of autoantigens [160]. Similarly, silica exposure is associated with autoimmune diseases like SLE and systemic sclerosis, with the inhalation of silica particles leading to chronic inflammation and immune system dysregulation [161].

## 7. Cytokine Therapy for Autoimmune Diseases

Cytokines play a crucial role in regulating the immune response and maintaining immune homeostasis. These signaling molecules mediate cellular communication to manage the equilibrium between proinflammatory and anti-inflammatory responses. However, the dysregulation of cytokine production contributes to the development and progression of several diseases, including autoimmune disorders and cancer. In autoimmune conditions, proinflammatory cytokines such as TNF-alpha, IL-6, and IL-1 are mostly increased. These cytokines accelerate the activation and proliferation of autoreactive T and B cells, causing the immune system to attack the body’s own tissues. For example, in rheumatoid arthritis, TNF-α and IL-6 are the main players causing inflammation and, consequently, joint destruction [162]. However, IL-1 promotes inflammatory processes in diseases like systemic lupus erythematosus [163]. Therefore, targeting cytokines has become an important strategy for developing novel therapeutics to treat various diseases. Neutralizing cytokines can have beneficial and harmful effects, and their use requires careful consideration of their complex role in disease pathophysiology.

## 8. Anti-TNF Therapy

TNF-α is an important proinflammatory cytokine released by various immune cells, including activated T and natural killer cells, monocytes, neutrophils, and macrophages [164]. Other cells, such as endothelial and fibroblast cells, also produce TNF-α [165]. However, monocytes and macrophages are the main sources of TNF-α in response to an inflammatory signal [166]. Nuclear factor-kB can directly attach to the promoter of the TNF gene, working in conjunction with nuclear factors associated with activated T-cells and AP-1 transcription factors to increase TNF mRNA levels [167].

In vitro, the upregulation of TNF results in the cytolysis and cryostasis of several tumor cell lines. In addition, high TNF levels can destroy the small blood vessels inside malignant tumors, leading to hemorrhagic necrosis in transplanted tumors. TNF affects the production of numerous other proteins, such as fos, myc, IL-1, and IL-6, and increases phagocytosis and cytotoxicity in neutrophilic granulocytes [168,169]. Increased TNF expression in the synovial fluid in RA and the progression of arthritis in a TNF-α-transgenic mouse model revealed that TNF plays a pathogenic role in chronic inflammation [170,171]. Treatment for patients with RA has undergone a radical change due to the effectiveness of anti-TNF therapy. Four monoclonal antibodies (mAbs) (golimumab, infliximab, certolizumab, and adalimumab) and etanercept have been approved for treating chronic inflammatory diseases, such as rheumatoid arthritis (RA), juvenile idiopathic arthritis (JIA), and ankylosing spondylitis (PsA) [172,173]. These antibodies do not influence lymphotoxin-alpha TNFR activity; however, they neutralize TNF receptor 1-2 (TNFR1 and TNFR2) activation by preventing TNF from attaching to its receptors. Etanercept and its biosimilars are soluble TNFR2 fusion proteins that prevent TNF and LT from attaching to their receptors [172].

Most inflammatory responses of TNF are mediated by TNFR1, expressed on the majority of nucleated cells, whereas the regulatory role of TNF is regulated by TNFR2, present on a small subset of cell types, including monocytes, Treg, and myeloid-derived suppressor cells [174,175]. TNFR2 expression plays a vital role in controlling immunological responses via signaling through Tregs to prevent the onset of inflammatory diseases. Furthermore, TNF inhibition may paradoxically increase the differentiation of Th1/Th17 cells and cause a dysregulated IFN response due to the regulatory nature of TNF, leading to autoantibody production and psoriasis after anti-TNF therapy [176,177]. Novel treatments have been explored to selectively activate TNFR2 or inhibit TNFR1 more effectively [174,178].

## 9. Anti-IL-1 Therapy

IL-1 is a dominant regulator of inflammation by regulating a diversity of innate immune mechanisms [179]. IL-1 induces extensive biological functions, including diverse acute-phase responses and lymphocyte-activating factors, and functions as a leukocytic pyrogen and a leukocytic endogenous mediator (LAF) [180]. In 1979, LAF was classified as IL in the Second International Lymphokine Workshop held in Switzerland. IL-1 was later discovered as an immunological mediator produced by macrophages acting on T and B lymphocytes [180,181]. The IL-1 family comprises two agonists, IL-1α and IL-1β. In contrast, the IL-1 receptor antagonist (IL-1Ra) is a receptor antagonist, and IL-1R type I (IL-1RI) and IL-1R type II (IL-1RII) are two different receptors. Both agonists (IL-1α, IL-1β) are proinflammatory cytokines directly related to innate immunity. Additionally, IL-1α and IL-1β share a binding site on the IL-1 receptor 1 (IL-1R1). However, some features distinguish IL-1α from IL-1β [182]. Unlike pro-IL-1β, which is released by macrophages and requires caspase-1 to be cleaved into its active form, mesenchymal cells constitutively express biologically active pro-IL-1α. An inflammasome composed of the nucleotide-binding domain (NLR) and leucine-rich-repeat (LRR) protein family is activated, causing the caspase-1-mediated cleavage of pro-IL-1β [183,184].

In contrast to other members of the IL-1 family, IL-1α is unique because it is constitutively present in the epithelial and mesenchymal cells of healthy individuals. In contrast, IL-1β is only produced under pathological conditions [184]. Additionally, unlike IL-1β, IL-1α is absent in the systematic circulation, indicating that the involvement of IL-1α in autoimmune diseases is limited to local tissues rather than systematic [185]. Therefore, IL-1β is closely related to various autoinflammatory diseases, including gout, familial Mediterranean fever, cryopyrin-associated periodic syndrome (CAPS), AOSD, and systemic JIA [186,187]. When IL-1α or IL-1β binds to IL-1R1, it forms a complex with another receptor (IL-1R3) to recruit myeloid differentiation primary response gene 88 (MYMYDD88). MYD88 recruitment initiates a proinflammatory kinase cascade that includes IL-1R-associated kinases (IRAKs), IκB kinase, and NF-κB [182,188]. IL-1Ra is a naturally produced IL-1 receptor antagonist that regulates the inflammatory activity by binding to its receptor, thus inducing a structural change that blocks the formation of a heterotrimeric complex with IL1R3 and disrupts the IL-1-dependent inflammatory response.

Rilonacept (IL-1R1 Fc protein), anakinra (recombinant IL-1 RA), and canakinumab (anti-IL-1 mAb) are protein-based therapeutics that are currently accepted as anti-IL-1 therapies [189]. All three drugs block the IL1 receptor, modulating the effects of IL-1α and IL-1β [182,190]. Moreover, novel drug design avenues, such as discovering potent small molecules to inhibit IL-1-mediated signaling, are currently being explored. Therefore, several small-molecule inhibitors are currently in pre-clinical trials and showing good inhibitory potential against IL-1 signaling.

IL-1 inhibitors are expected to be clinically beneficial in treating incurable autoimmune disorders, such as SLE and systemic sclerosis, and in managing increased proinflammatory reactions, such as macrophage activation and cytokine release syndromes [182,191]. Therefore, IL-1 is potentially a desirable molecular target for treating various diseases, including malignant tumors, metabolic syndromes, and autoimmune, infectious, autoinflammatory, and autoimmune diseases [192,193].

### 9.1. IL-6

IL-6, a pleiotropic cytokine, is released by several cell types during infection, cancer, and inflammation. IL-6 was first discovered as a “B cell differentiation factor” or “B cell stimulation factor” produced by T-cells [194]. Anti-IL-6 therapy is unsuccessful in treating patients with multiple myeloma despite having a vital role in the maturation of B cells [195]. Tocilizumab, the first monoclonal antibody to inhibit IL-6R, demonstrated considerable therapeutic advantages in patients with RA and had better efficacy than adalimumab [196]. Depending on the capacity to control systemic hyperinflammation, tocilizumab is now used to treat RA, JIA, giant cell arteritis (GCA), AOSD, and cytokine release syndrome (CRS) [194]. Anti-IL-6 therapeutics have recently been studied as a potential treatment for systemic sclerosis, SLE, and neuromyelitis optica (NMO) [197,198]. In physiological states, IL-6 plays a significant role in regulating M2-state macrophage differentiation and osteoclast genesis via the enhanced expression of the receptor activator of nuclear factor kappa-Β (RANK) ligand on osteoblasts and is a key inducer of the acute-phase response and infection defense in the liver [199]. IL-6 regulates the expression of FOXP3, RORC, and IL-23R in inflammatory conditions, which is essential for Th17 differentiation [199,200,201]. Additionally, IL-6 is a key molecule in B-cell maturation and follicular helper T-cell development [202].

IL-6 signaling is mediated via an IL-6R and glycoprotein (gp130) complex. Multiple signaling pathways, such as mitogen-activated protein kinase (MAPK), phosphoinositide 3-kinases (PI3K), Janus kinase (JAK)-signal transducer, activator of transcription (STAT), and YES-associated protein 1, are successively activated by the dimerization of gp130. YES-associated protein 1, upon nuclear translocation, regulates gene transcription, inducing proliferation, cell growth, and inflammation [203,204]. The strategy to inhibit IL-6-mediated signaling includes the inhibition of IL-6, IL-6R, STAT3, JAK, and gp130. The therapeutic potential of STAT3 and gp130 are promising; however, their inhibition can induce side effects [205,206].

### 9.2. Type 1 Anti-IFN Alpha Therapy

Over 60 years ago, Isaacs and Lindenman identified IFN-I and demonstrated that IFN-Is have antiviral activity. Type 1 IFNs were first discovered as soluble antiviral factors and include IFN-α, IFN-β, IFN-ε, and IFN-ω [207]. IFN-Is cytokines are crucial for regulating immune response, recognizing tumor cells, and stimulating T-cell responses [208]. IFN1-mediated signaling is initiated upon binding to a type 1 IFN receptor, composed of IFN-alpha and -beta receptor subunits 1 and 2 (IFNAR1 and IFNAR2) [209,210]. IFN-α plays a significant role in the pathophysiology of autoimmune disorders, particularly in SLE, as a clinical trial suggested an increase in IFN-1 expression in SLE [211].

All cell types have IFN-I receptors. However, the key cellular source is plasmacytoid DCs (pDCs), which are key elements of the innate immune system [212]. TLRs 7 and 9 in plasmacytoid DCs (pDCs) interact with viral RNA and DNA to recruit MYD88, further activating TRAFs and IRAKs. The activation of TRAFs and IRAKs causes IFN regulatory family (IRF) 7 translocation and triggers IFN transcription [207,213]. IFNAR1 and IFNAR2 stimulate JAK1 and TYK2, respectively, in response to type 1 IFN interaction, leading to STAT1 and STAT2 phosphorylation and translocation to the nucleus. The expression of IFN-stimulated genes is induced by STAT1/STAT2, along with IRF9 [207]. IFN stimulates myeloid DCs and B and Th1 cells while suppressing Tregs to increase the inflammatory response in SLE [213].

The pathophysiological role of IFN-α in various systematic autoimmune diseases (SADs), including SLE, has contributed to developing targeted immunotherapy against IFN-I. Two monoclonal antibodies, sifalimumab and rontalizumab, were designed to neutralize IFN-α and failed to suppress SLE [214,215]. Although the function of IFNs other than IFN-α is unclear, the defective therapeutic effect on SLE may be explained by the suppression of IFN-α alone while leaving other active forms, such as IFN-κ and IFN-β. Recently, anifrolumab, a human monoclonal antibody (mAb) targeting IFNAR1-mediated signaling, inhibiting the activity of all type 1 IFNs, was approved to treat SLE [216].

Several novel treatment strategies to inhibit type 1 IFNs have been studied. Drugs have been developed to target pDCs and signaling molecules such as TLR, MyD88, and IRAK4 to decrease the production of type 1 IFNs [213].

### 9.3. IL-17

The family of proinflammatory cytokines known as IL-17 is generated by Th cells in response to IL-23 activation [217]. Additionally, CD8^+^ T-cells (Tc17), natural killer T (NKT) cells, type 3 innate lymphoid cells (ILC3), and neutrophils produce IL-17 [218]. Th17 cells provide host defense against fungi and bacteria, neutrophil recruitment, tissue repair, and innate immunity in the physiological state [219,220]. IL-17A induces autoimmunity under pathological conditions by working with other inflammatory chemokines, such as TNF [221]. Animal models of inflammatory arthritis and neuroinflammation reveal that Th17 cells play a major role in disease pathogenesis [222].

Inhibiting FOXP3 and stimulating signal transducer and activator of transcription 3 (STAT3) and RAR-related orphan receptor C (RORC) in the presence of IL-1 β, TGF, IL-6, and naive CD4^+^ T-cells give rise to Th17 cells [201]. Then, IL-23 stabilizes pathogenic Th17 cells to promote their survival and proliferation [223]. The adaptor protein Act1 is recruited by the IL-17A-specific cytoplasmic domain known as similar expression to fibroblast growth factor (SEF) of IL-17R subunits A (IL-17RA) and C (IL-17RC), which then ubiquitinates TNF-receptor-associated factor (TRAF) 6 and activates the nuclear factor-κB (NF-κB), mitogen-activated protein kinase (MAPK), activator protein 1 (AP1), and C/EBP transcription factors [224,225].

Studies on animals and humans have demonstrated the IL-23/IL-17 association as a potential therapeutic target for various autoimmune diseases, including ankylosing spondylitis (AS), psoriatic arthritis (PsA), and RA [221,226,227]. Secukinumab and ixekizumab are two mAbs for IL-17A and IL-17RA that are now used in anti-IL-17 therapy. Anti-IL-17 therapies are expected to dramatically improve symptoms in patients with psoriasis, AS, and PsA [170]. Surprisingly, this strategy did not demonstrate exceptional therapeutic results in TH17-mediated diseases, such as RA [210,228]. IL-17A-mediated diseases are difficult to treat due to their heterogeneous nature and the variable pathogenic role of IL-17A.

### 9.4. IL-23

IL-23 is a heterodimeric proinflammatory cytokine released by macrophages upon activation and DCs [211]. IL-23 is constituted of the p40 subunit, which is identical to IL-12, and the novel cytokine subunit p19, which is unique to IL-23 [212]. Despite having a similar structure, IL-23 plays a more significant role in the autoimmune disorder etiology than IL-12 [213]. IL-23 upregulates the expression of IL-23R, induces effector genes, maintains the expression of Th17-specific genes, and stabilizes the pathogenic characteristics of Th17 cells [212,214].

IL-23 binds to IL-12R1 and IL-23R receptors, which are associated with tyrosine-protein kinase (TYK2) and Janus kinase 2 (JAK2). Both TYK2 and JAK2 activation trigger the nuclear transportation and phosphorylation of signal transducer and activator of transcription (STAT3) [215]. IL-23-mediated signaling can be blocked by targeting IL-12p40 or IL-23p19. Ustekinumab is a monoclonal antibody that inhibits both IL-12 and 23 (IL12/23). In contrast, risankizumab, tildrakizumab, mirikizumab, and guselkumab solely target IL-23. Additionally, IL-23 inhibition is effective against autoimmune inflammatory diseases, such as psoriatic arthritis (PsA) and psoriasis [216]. Although IL-23 played an obvious pathological role in ankylosing spondylitis (AS) clinical models, it was ineffective in treating AS. The variation in this result could be due to its different roles, functioning in a tissue- and time-dependent manner, in human autoimmune disorders [226]. Ustekinumab successfully treated Crohn’s disease and improved the clinical outcomes of patients with Crohn’s disease. Recent autoimmune disease treatment targeting IL-23 includes preventing the interaction of IL23 with its receptor (IL-23R) or disrupting downstream signaling using antibodies or small-molecule inhibitors.

## 10. Alternative Therapeutic Approaches Targeting Immune Cells

Autoimmune diseases are known to cause a breakdown in immune tolerance, leading to the production of autoreactive B and T-cells and autoantibodies that damage organs. These diseases include systemic lupus erythematosus (SLE), rheumatoid arthritis (RA), multiple sclerosis (MS), and type 1 diabetes, among others. In this context, alternative therapeutic approaches targeting T and B cells offer a broad spectrum of treatment options for autoimmune diseases alongside conventional therapies.

B cells play a significant role in autoimmunity by producing autoantibodies, enhancing CD4+ T-cell responses, secreting proinflammatory cytokines, and depleting specific cell types [217,218,219]. They can produce cytokines such as IL-6, TNF, IL-10, and IFN-g, impacting autoimmune pathology and contributing to the cytokine environment that causes T-cell polarization [220]. Rituximab, one of the first FDA-approved monoclonal antibodies (mAb) targeting CD20-positive B cells, is used in treating non-Hodgkin’s lymphoma [220,221]. B-cell depletion therapies have shown effectiveness in various autoimmune diseases, highlighting the significance of B lineage cells in the pathogenesis of these conditions [222,223]. However, monoclonal antibodies have limitations in completely depleting B cells within inflamed tissues and lymphatic organs. CAR T-cells offer a potential advantage in targeting cells with low antigen expression [224,225]. Maintaining a balance between the effectiveness and safety of CAR T-cells is crucial, requiring careful dosing to avoid toxicities like cytokine release syndrome (CRS) and neurotoxicity in patients [217].

Chimeric antigen receptor (CAR) T-cell therapy has emerged as a promising method for treating autoimmune diseases. CAR T-cell therapy involves engineering T-cells to express CARs that target specific antigens on B cells [226]. In autoimmune diseases like SLE and dermatomyositis, CAR T-cells have shown effectiveness in rapidly and sustainably depleting B cells [229]. CAR T-cells operate by identifying and binding to antigens on B cells, activating and subsequently destroying these target cells. This approach leverages the intrinsic ability of T-cells to penetrate tissues and establish high-affinity binding to specific targets. The therapy consists of an extracellular antigen-recognition domain derived from T-cell and antibody intracellular activation domains, ensuring the proper activation and expansion of CAR T-cells [227]. CAR T-cell-based therapy shows potential for curing B-cell malignancies, with similar outcomes expected for autoimmune diseases [228]. Future research, such as advancements in genetic engineering, is needed to enhance CAR T-cell-based therapy, improving safety profiles and expanding treatment to a broader range of autoimmune diseases.

## 11. Conclusions

In conclusion, autoimmune diseases result from the breakdown of immune tolerance, which can be caused by various predisposing factors, such as genetic susceptibility and environmental triggers. The mechanisms of immunity, including central and peripheral tolerance, play a crucial role in maintaining self-tolerance and preventing autoimmunity. Understanding these mechanisms and the factors contributing to tolerance breakdown is essential for developing targeted immunotherapies for autoimmune diseases. These therapies aim to modulate the immune system and restore self-tolerance, thus leading to improved disease management and better quality of life for the affected individuals. Further research is necessary to identify novel immunotherapy targets and optimize the efficacy and safety of the existing treatments.

## Abbreviations

ADP, adenosine diphosphate; AIRE, autoimmune regulator; ATP, adenosine triphosphate; B reg, regulatory B cell; CTLA-4, checkpoint protein; CTLA-4, cytotoxic T-lymphocyte-associated protein 4; DC, dendritic cell; FOXP3, forkhead box P3; GWAS, genome-wide association study; HLA-DRB1, human leukocyte antigen-DRB1; IFN, interferon; IL, interleukin; MHC, major histocompatibility complex; mTEC, medullary thymic epithelial cell; pTregs, peripheral derived regulatory Tregs; RA, rheumatoid arthritis; SLE, systemic lupus erythematosus; TCR, T-cell receptor; Teff, effector T-cell; TGF, transforming growth factor; Th, T helper; Th3, TGF-producing type 3 helper T-cells; TLR, toll-like receptor; Tr1, IL-10-producing type 1 regulatory T; Treg, regulatory T-cell; tTregs, thymus-derived Tregs; UV, ultraviolet.
